# Mistletoe viscin: a hygro- and mechano-responsive cellulose-based adhesive for diverse material applications

**DOI:** 10.1093/pnasnexus/pgac026

**Published:** 2022-03-16

**Authors:** Nils Horbelt, Peter Fratzl, Matthew J Harrington

**Affiliations:** Department of Biomaterials, Max Planck Institute of Colloids and Interfaces, Potsdam 14424, Germany; Department of Biomaterials, Max Planck Institute of Colloids and Interfaces, Potsdam 14424, Germany; Department of Biomaterials, Max Planck Institute of Colloids and Interfaces, Potsdam 14424, Germany; Department of Chemistry, McGill University, 801 Sherbrooke Street West, Montreal, Quebec H3A 0B8, Canada

**Keywords:** bioadhesive, cellulose, films, mechanoresponsive

## Abstract

Mistletoe viscin is a natural cellulosic adhesive consisting of hierarchically organized cellulose microfibrils (CMFs) surrounded by a humidity-responsive matrix that enables mechanical drawing into stiff and sticky fibers. Here, we explored the processability and adhesive capacity of viscin and demonstrated its potential as a source material for various material applications, as well as a source for bioinspired design. Specifically, we revealed that viscin fibers exhibit humidity-activated self-adhesive properties that enable “contact welding” into complex 2D and 3D architectures under ambient conditions. We additionally discovered that viscin can be processed into stiff and transparent free-standing films via biaxial stretching in the hydrated state, followed by drying, whereby CMFs align along local stress fields. Furthermore, we determined that viscin adheres strongly to both synthetic materials (metals, plastics, and glass) and biological tissues, such as skin and cartilage. In particular, skin adhesion makes viscin a compelling candidate as a wound sealant, as we further demonstrate. These findings highlight the enormous potential of this hygro- and mechano-responsive fiber-reinforced adhesive for bioinspired and biomedical applications.

Significance Statement Biological materials constitute raw materials as well as role models for development of next generation plastics, composites, and glues. Here, we demonstrate the potential of mistletoe viscin as both a source material and a source of bioinspiration. Viscin is a fiber-reinforced adhesive produced in mistletoe berries, crucial for seed dispersal of this parasitic plant. Our findings indicate that viscin's unique hierarchical structure, consisting of CMFs embedded in a humidity sensitive adhesive matrix, enables easy processing into 2D/3D frameworks and free-standing transparent films. This is achieved through simple mechanical manipulation under ambient humidity conditions. Furthermore, viscin's versatile adhesive properties allow it to stick to almost any synthetic or biological surface, providing possible applications as biomedical skin coverings or wound sealants.

## Introduction

Adhesives are a technically and biomedically important class of materials that perform essential functions as engineering sealants, surgical glues, and adhesive tapes. In recent years, scientists and engineers have looked to nature to address specific deficiencies in man-made adhesives, including reversible adhesion and adhesion under wet conditions (1–4). Indeed, natural selection has resulted in the evolution of numerous versatile adhesives with properties unmatched in current synthetic adhesives. Striking examples include the wet adhesives produced by mussels and velvet worms (5, 6), as well as the reversible dry adhesion exhibited by geckos and insects (7, 8). Elucidation of the chemical and physical principles underlying adhesion in these systems has led directly to the invention and application of synthetic adhesives with both technical and biomedical functions (2, 9, 10). Along these lines, recent work has identified mistletoe viscin as an exciting model system of a fiber-reinforced adhesive that combines strong adhesion with exceptional mechanical properties and facile processability (11). Here, we more deeply explore the mechano- and hygro-responsive adhesive properties of this cellulosic bioadhesive, and its potential for bioinspiration and biomedical applications.

**Fig. 1.**
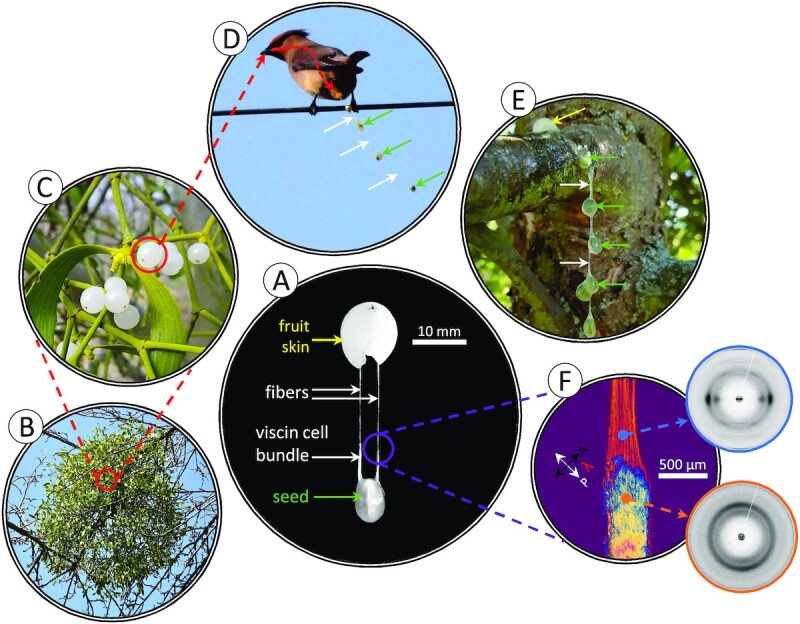
Adhesive mistletoe viscin fibers. (A) A mistletoe seed manually extracted from a *V. album* berry showing the viscin cell bundle (VCB) and the beginning of fiber formation. (B) *Viscumalbum* plant in winter growing on a deciduous host. (C) Closer view of *V. album* plant showing translucent white berries. (D) A Japanese waxwing (*Bombycilla japonica*) leaving a chain of mistletoe seeds connected via adhesive viscin fibers during defecation. White arrows: thin, barely visible viscin fibers. Green arrows: seeds. (E) A chain of mechanically isolated *V. album* seeds manually deposited on an apple tree branch. Yellow arrow: fruit skin. (F) Polarized light microscopy (PLM) image of VCB and fiber, as well as WAXS patterns from both tissues, highlighting the high alignment of cellulose during mechanical fiber pulling. The photo in panel (D) was adapted and used under a CC BY 2.0 license from https://www.flickr.com/photos/conifer/13083153535/in/album-72157600077948977/, https://creativecommons.org/licenses/by/2.0.

The European mistletoe (*Viscum album* L.) is an aerial hemiparastic plant species with a long history of human cultural relevance dating back at least to the time of the ancient Greeks (12, 13). Mistletoe plants grow on the branches of various host trees and are spread by birds who eat the white berries in winter and spread the seeds (Fig. 1) (14–16). Seeds are surrounded by a sticky mucilaginous tissue known as viscin, which is comprised of hierarchically organized cellulose microfibrils (CMFs) embedded in a hygro (humidity)-responsive matrix (Fig. 1) (11, 17). A previous compositional study of the viscin tissue from *V. album* indicated that there are approximately equal parts of cellulose and various hemicelluloses (enriched in arabinose, mannose, and galactose), with a small proportion of pectins based on detected uronic acid content (∼2 dry wt%) (17); however, there are few compositional details beyond this study. Typically, mistletoe berries quickly pass through the digestive tract of birds and are excreted as a sticky viscin fiber containing several seeds that then become adhered to tree branches, enabling germination and fusion with the host plant (Fig. 1) (14). Alternatively, the sticky seeds may become adhered to the beak of the bird, after which they are spread by dislodgement onto a tree branch (15). Unlike most other biological adhesives, mistletoe viscin combines strong adhesion with the ability to be rapidly processed via simple mechanical drawing into stiff, yet flexible fibers of up to 2 m length that are reinforced by highly aligned CMFs (Figure S1, Supplementary Material) (11). Notably, a previous study showed that viscin fiber stiffness is highly tunable based on the relative humidity (RH) in the local environment—near 0% RH, fiber stiffness up to 20 GPa was measured, while at RH close to 95%, fiber stiffness is reduced to around 300 MPa (11). Additionally, above 50% RH, the fibers exhibited an ability to flow under strain, whereas at low RH the fibers exhibited ultimate strain values of less than 2%. This hygroresponsive behavior is fully reversible; yet, in spite of the humidity dependent mechanical variability and biological origin of the fibers, the mechanical properties at a given RH were shown to be reproducible (11).

Recent in-depth structure–function investigations of the fiber formation process (11) provide insights into the nanoscale mechanism, highlighting the crucial importance of the swellable hygro- and mechano-responsive matrix in the processability of mistletoe viscin. Indeed, these studies suggest that the noncellulosic matrix material surrounding the CMFs responds to changes of the RH, rapidly absorbing water vapor above ∼50% RH, allowing CMFs to slide past one another at multiple length scales and align under tensile load. Yet, upon drying, the matrix functions as a strong cement binding the highly aligned CMFs together, resulting in impressive tensile stiffness (11). Insights into the hygro- and mechano-responsive processability of the viscin fiber material emerging from these studies may provide design principles relevant for ongoing efforts to produce cellulosic composites (18–21). However, the combination of strong adhesion, mechanical integrity, and ease of processability also make mistletoe viscin a distinctive multifunctional model system for inspiration of next-generation technical and biomedical adhesives. Here, we demonstrate that viscin can be processed into more complex architectures and structures beyond simple fibers and investigate the adhesive properties of the viscin tissue and its potential as a biomedical sealant. Our findings demonstrate the enormous potential of this system for bioinspired design and applications.

## Results

### Humidity-activated fusion of viscin fibers

**Fig. 2.**
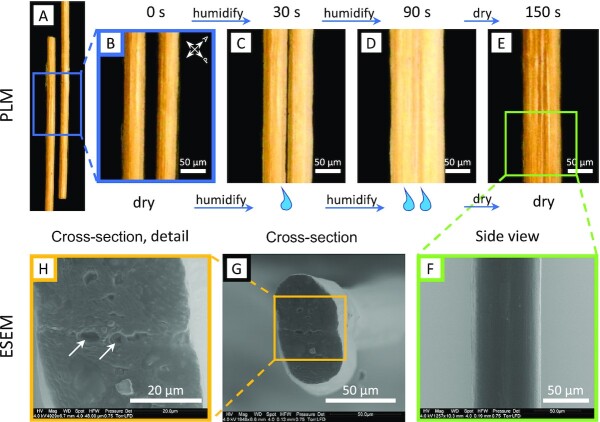
Hygro-activated fusion of viscin fibers. (A) PLM image of 2 loose ends of dried viscin fibers oriented at 45° to the polarization filters. (B) Detail of (A). (C) Fibers were brought into contact. Slightly swollen fibers after 30 s exposure to saturated water vapor (RH ∼ 100%). The interface between the 2 fibers is still visible. (D) Fibers at maximum swelling after 90 s of rehydration. The fibers fuse along the contact zone and the interface is no longer visible. (E) Dried fibers viewed 60 s after maximum swelling (total experimental time of 150 s, revealing that the diameter of the fused fibers is reduced dramatically, but the interface between fibers is not observable. (F) ESEM image showing a side view of the fused fibers. (G) ESEM image of a fused fiber cross-section. Scale bar: 50 µm. (H) Detail of (G) revealing that the interface between the fused fibers is essentially lost. Arrows: voids due to possible inclusions at the former fiber–fiber interface.

The ability of viscin fibers to self-adhere is relevant to their biological function, in which several berries will produce long chains of seeds that adhere to tree branches (Fig. 1D). However, this phenomenon does not require the seed's passage through a bird's digestive system. This can be easily demonstrated by the mechanical isolation and mixing of fresh *V. album* seeds, which leads to a similar seed chain (Fig. 1E). Importantly, self-adhesion only occurs in the hydrated state and was not observed between dried fibers when brought into contact. However, it was noticed during the course of our studies that 2 dried fibers held together between fingers will fuse with one another, possibly using the humidity from the skin to initiate the adhesive interaction. To further investigate the ability to reactivate the self-adhesive properties of the dried fiber matrix, 2 pieces of stiff fiber were brought into contact in the presence of increased RH. As the RH was raised above ∼40%, the hygroscopic fibers absorb water from the air, swelling slightly (as previously reported (11)), and when brought into contact, begin to adhere and deform at the interface (Fig. 2C, see Video S1 for the entire fusion process). Further raising the humidity by exposing the fiber ends to saturated water vapor (∼100% RH) leads to rapid and intense swelling of the fibers with deformation along the interface between the fibers (Fig. 2D). Upon reducing RH back below 40%, the fibers begin drying, but remain physically fused to one another, exhibiting loss of a clear interface (Fig. 2E and F), as observed in environmental scanning electron microscopy (ESEM) imaging (Fig. 2G and H). In light of previous studies (11), these observations suggest that the swellable matrix may also mediate self-adhesion. Considering that the fused fiber ends had been cut with a razor blade, this could also be considered an example of a humidity-activated intrinsic self-healing response.

### Humidity-based contact welding of complex architectures from viscin fibers

**Fig. 3.**
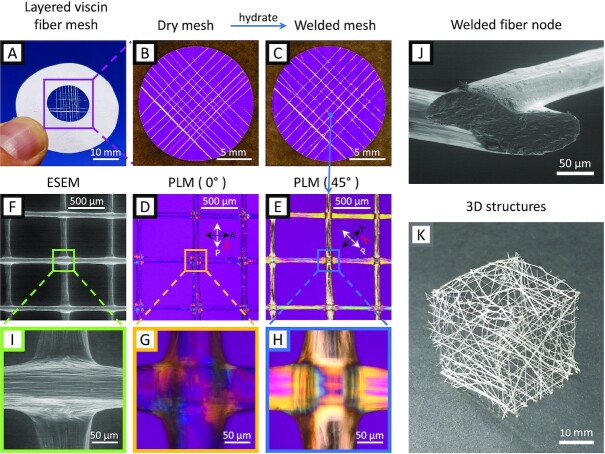
Structures made from viscin fibers. (A) A mesh of viscin fiber segments arranged in a cross-wise pattern glued on a cardboard frame. (B) PLM overview image of a dry viscin mesh. (C) PLM image of the contact welded viscin fiber mesh after brief rehydration. (D) ESEM image of welded fiber nodes. (E) PLM image of welded fiber nodes. (F) PLM image of welded fiber nodes at an angle of 45° with respect to the polarization filters. (G) ESEM detail of a fiber node showing the distorted viscin on the fiber surface around the node edges. (H) and (I) PLM details. (J) An oblique view on the cross-sections of 2 fused fibers crossing at an angle of ∼90° and sectioned at an angle of ∼45° close to the node. (K) A hollow cube formed of premanufactured 2D meshes fused by rehydration.

As a next step, we harnessed the humidity-initiated fusion of stiff viscin fibers to construct 2D and 3D structures using a simple hygro-responsive welding method. Viscin from a single berry can produce a fiber of ∼2 m in length. Cut pieces of a single mistletoe fiber were arranged in a desired configuration in the dry state (e.g. layered mesh) and exposed briefly to water vapor and then allowed to air dry (Fig. 3A–C). The physical contact welding of the fibers was confirmed using ESEM and PLM (Fig. 3D–F), showing a flattening and welding at the junction of 2 fibers with a loss of the interface and a local distortion of the cellulose orientation (Fig. 3G–J). This process indicated that the hygroscopic viscin fibers can be contact welded using only elevated moisture at room temperature to join the pieces. This is a remarkable behavior for biopolymeric fibers with a stiffness in the dry state of more than 14 GPa (i.e. exceeding that of Nylon by more than 3-fold (22)). 2D structures can be expanded into multilayer architectures similar to the additive manufacturing of 3D printed objects by simply layering multiple fibers on top of one another, creating more complex, and presumably more stable junction points (Figure S2, Supplementary Material). Moreover, dried viscin fibers can be used to construct 3D objects by a stepwise premanufacturing of several 2D mesh structures, which can later be assembled into the desired 3D shape and welded by local rehydration along the junction zones (Fig. 3K).

### Processing of free-standing viscin films

**Fig. 4.**
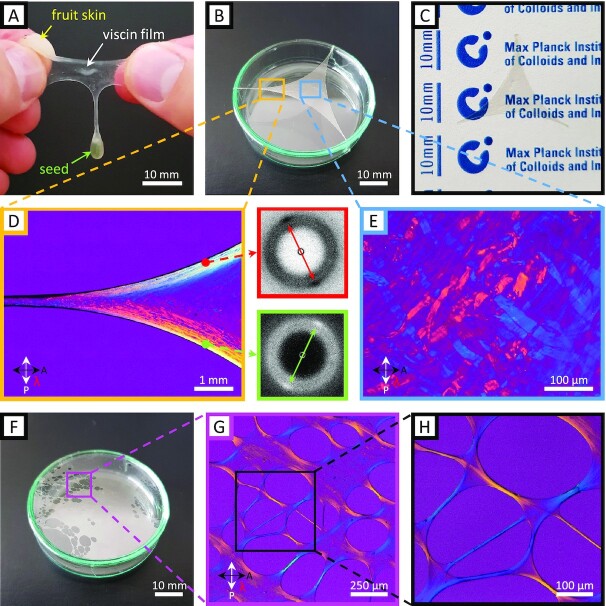
Viscin film properties examined with PLM and WAXS. (A) Image of a freshly formed and flexible viscin film drawn from a compressed berry of *V. album*. (B) Free-standing viscin film drawn into a triangular shape and glued to the edge of a petri dish, making use of the natural adhesive properties. (C) A dried viscin film is dimensionally stable and highly transparent. (D) PLM image showing details of (B) revealing the cellulose orientation along the contours of a film. Cellulose orientation was also confirmed with wide-angle X-ray diffraction shown in the adjacent diffraction pattern from 2 selected points along the film contour. The arrows in the diffraction patterns mark the distinct equatorial cellulose diffraction spots. (E) PLM image of the film center from (B) where viscin cells are found to be randomly oriented and mostly unstretched. (F) A viscin film, which locally collapsed into a porous structure. (G) PLM image showing a detail of a porous region from (F). (H) Detail from (G) showing that the film locally collapsed into small fibers. The cellulose is highly aligned along the fiber directions and the pore contours as indicated by the polarization colors.

It was observed during our studies that viscin not only forms fibers, but also films under appropriate processing conditions. This can be most simply demonstrated by rubbing fresh hydrated viscin tissue between fingers and then slowly separating the fingers (Fig. 4A). Due to the adhesion of viscin to the fingertips, it can be stretched biaxially forming a film (see Figure S3 [Supplementary Material] for a more detailed description). Further stretching results in the eventual collapse of the films into a fiber. In a more controlled manner, freestanding films of mechanically isolated viscin were formed by first fixing short and thick strands on 2 different points on a substrate (here, the edges of a petri dish), and then simply pulling in an opposing direction and fixing to a third point (Fig. 4B, see Figure S4 [Supplementary Material] for a more detailed description). The resulting transparent films, like the fibers, are hygro- and mechano-responsive, flowing under applied load when wet, stiffening considerably when dried, but notably retaining their integrity and shape (Fig. 4C). PLM imaging of films reveals orientation of CMFs along the local contours of the anchoring points (Fig. 4D). This orientation was verified with wide angle X-ray scattering (WAXS), showing that cellulose is aligned along the apparent stress fields. In the middle of films, in contrast, numerous randomly oriented unstretched viscin cells were observed (Fig. 4E)—which could ostensibly supply further film extensibility in the wet state and also resist failure of the films under drying stresses. A viscin film, which is excessively stretched will fail eventually; however, failure is not characterized by catastrophic rupture of the film, but rather is initiated by a localized collapse of the film into a porous architecture (Fig. 4F). The pores are connected via thin fiber segments with highly oriented cellulose microfilaments along the contours of pores as indicated by PLM, ensuring the mechanical integrity of the remaining film (Fig. 4G and H).

### Versatile adhesion of viscin on synthetic and biological surfaces

**Fig. 5.**
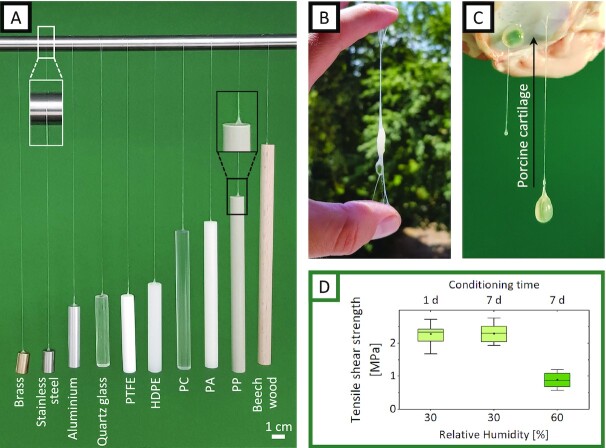
The multimaterial adhesive properties of viscin. (A) Cylinders from 10 selected materials with different surface chemistries are supported by a viscin fiber, each attached to the top surface of the cylinder and a laboratory stand. Cylinder diameter: 1 cm and cylinder weight: 10 g. (B) A viscin fiber adhered between 2 fingers supporting the seed. (C) A total of 2 *V. album* seeds adhering to porcine cartilage. A total of 1 seed is directly attached to the cartilage via the hydrated viscin layer surrounding the seed. The other seed is connected via a freshly drawn viscin fiber. (D) Adhesion strength extracted from lap shear tests performed on wood surfaces at low and high RH and at different time points.

Based on the observation that hydrated viscin fibers adhere to themselves, to tree branches, and to fingertips (and other materials observed during our investigations, such as glass slides, tweezers, and lab bench surfaces), we further explored the versatility of the viscin adhesive on a wide range of surfaces presenting different chemistries including metals (brass, aluminum, and stainless steel), glass, mica, thermoplastics (polytetrafluoroethylene (PTFE), high density polyethylene (HDPE), polycarbonate (PC), polyamide (PA), polypropylene (PP)), and wood. For these selected materials, the viscin from a single berry was able to adhere to a small area and support the weight of 10 g of each material (Fig. 5A). Considering that the typical weight of a hydrated seed is on the order of 0.2 g, the dried viscin can withstand loads at least 50x higher than required based on the assumed natural function and likely more. The adhesive strength of viscin was measured between 2 wood surfaces using a standard lap shear test. Values of more than 2 MPa were measured at 30% RH with fresh and week-old adhesives, while values decreased below 1 MPa when measured at 60% RH. The observed humidity-dependent mechanics are completely consistent with previous measurement of viscin fiber mechanics, which showed a large decrease in sample stiffness above 45% RH (11).

**Fig. 6.**
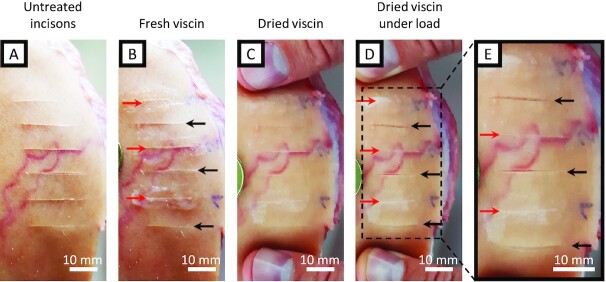
Image series of an artificial wound treatment with viscin. (A) Parallel cuts were made with a razor blade on porcine skin. (B) A total of 3 incisions were selected as reference (black arrows) while the remaining incisions were used for viscin treatment (red arrows). Freshly isolated viscin from 1 berry each was spread over the selected incisions. (C) Applied viscin after drying. (D) Dried viscin sealant loaded perpendicular to the incisions. (E) Detail from (D) showing the opened reference incisions under load while the dried viscin sealant keeps the incisions sealed and closed.

In addition to the materials tested above, it was demonstrated that fresh viscin could adhere firmly to human skin and nonliving porcine cartilage. The ability of mistletoe viscin to adhere to biological tissues coupled with its biomolecular composition makes it a compelling candidate as a wound sealant or biomedical adhesive. Indeed, there are reports of mistletoe viscin being used both as a glue for capturing birds (known as birdlime) and wound dressing in ancient times (reviewed in Tubeuf 1923, p. 41 (13)), and commercially available nitrocellulose-based sealants for small cuts can be found at most drugstores (e.g. Filmogel, Laboratoires Urgo, France). To investigate the potential of this natural fiber-reinforced adhesive as a wound sealant, incisions were made in porcine skin (nonliving) using a razor blade (Fig. 6A). The incisions were sealed by spreading native isolated viscin tissue over the cut (Fig. 6B) and allowing it to dry (Fig. 6C). The silky, glossy viscin sealant remained attached upon drying, and even when a load was applied to the skin, the cut remained sealed, while nearby unsealed incisions opened easily (Fig. 6D and E). Tested on live human skin (no incisions inflicted), such a viscin sealant remained firmly attached for a period of at least 3 days. The sealant always retained a minor tack on its surface, but remained flexible, allowing free movement when performing everyday tasks and was even resistant to brief rinsing with water. To remove the tissue seal, friction could be used by simply rubbing the sealed area.

**Fig. 7.**
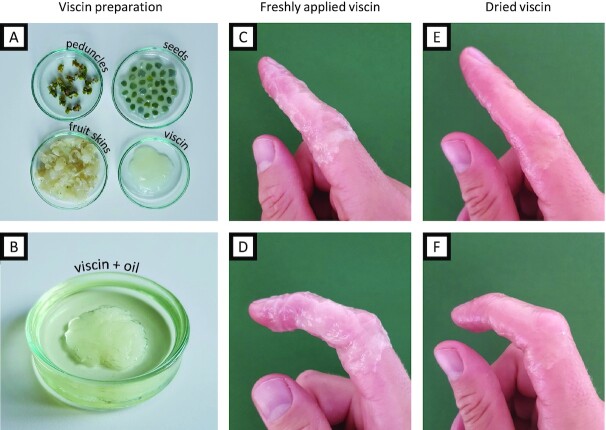
Preparation and application of viscin skin covering. (A) Mechanically separated fraction of the *V. album* berries: peduncles, seeds, skins, and flesh and viscin. (B) Viscin submerged in walnut oil. (C) Translucent, milky viscin coating from (B) freshly applied to human skin, covering a finger. (D) The freshly applied viscin allows free movement of the covered finger. (E) The viscin coating dried into a thin transparent film. (F) The dried flexible viscin coating still allows free movement of the finger without failure despite intense stretching and compression, e.g. around the knuckles.

Native viscin performed surprisingly well when used as a tissue sealant, considering this is not the evolved function. However, the intrinsic property of viscin to stick to various surfaces and the ability to instantly form fibers made it delicate to handle and complicated the precise application of the seal onto only the designated area. Therefore, we explored ancient recipes dating to several hundred years BCE for making viscin birdlime and coatings, which mention the use of natural oils as an additive (Theophrastus (371–287 BC): *De Causis Plantarum* and Pliny (AD 24–79): *Naturalis historia*, reviewed in Tubeuf 1923, p. 50 (13)). In a very simplistic approach, mechanically isolated viscin from multiple berries was submerged in walnut oil or olive oil for a few minutes (Fig. 7A and B). The oil-treated viscin exhibited reduced tack compared to native viscin and showed reduced propensity for fiber formation, simplifying processing. However, it still exhibited notable adhesion to human skin and improved mechanical coherence. The oil-processed viscin feels smooth and silky and can be kneaded like a dough, stretched easily into stable films, and applied to the skin, where it can be further redistributed over the designated area (Fig. 7C and D). Within only a few minutes, even thickly applied viscin dries into a smooth transparent coating (Fig. 7E). Because the oil-treated viscin exhibits reduced adhesive tack, one can grab or touch things without sticking to the surface. Moreover, the coating is highly flexible (perhaps due to the skin humidity) and does not restrict any movements (Fig. 7F). Indeed, it is practically imperceptible on the skin. Similar to the native viscin, defects can be repaired by adding further material or by locally rehydrating the coating, which enables further manipulation consistent with capacity of the material to exhibit contact welding under ambient conditions. To wrap an entire finger with a viscin coating as presented in Fig. 7(C) and (D) requires the viscin of only about 10–15 berries (for reference, a single mature plant typically produces thousands of berries in 1 season).

## Discussion

### Reversible hygro- and mechano-responsive adhesive

Here, we demonstrated the versatile processability of the mistletoe viscin adhesive, including the capacity for humidity-initiated contact welding to form 2D and 3D architectures, formation of free-standing films revealing dynamic and adaptive reorientation of cellulose, strong adhesion to a broad range of surface chemistries and finally, the ability to act as a biogenic adhesive and tissue sealant that is effective on mammalian skin. These properties result from the intrinsic combination of a hygro-responsive versatile adhesive mechanically reinforced by stiff CMFs. The resulting multifunctionality highlights the enormous potential for bioinspiration including potential biomedical applications.

As noted, dry viscin fibers are highly flexible, with stiffness values far exceeding those of most standard thermoplastics, with the exception of ultrahigh-performance technical plastics (11, 22–24); However, unlike petroleum-based thermoplastics, viscin fibers are produced and processed under sustainable and environmentally friendly conditions and are additionally biorenewable, biodegradable, and likely biocompatible. It is worth nothing that given the humidity dependence of their processing and properties, viscin fibers are better categorized as hygroplastics than thermoplastics (25). Indeed, at RH above ∼50%, the hygroscopic matrix rapidly absorbs moisture from the atmosphere, which converts it from a strong cement to a mechano-responsive sacrificial binder, allowing the CMFs to flow past one another under mechanical load, enabling elongation of the tissue into meter-long fibers (11). Additionally, the hydrated matrix also enables viscin fibers to adhere to one another, as well numerous other surfaces. From an evolutionary perspective, the propensity of viscin from multiple berries to stick to one another is advantageous in order to create a network of fibers in the bird's gut, that when released, presumably increases the likelihood of adhesion to branches, and thus, seed propagation and germination. However, we demonstrated that the hydration-dependent tendency of the viscin tissue to adhere indiscriminately to itself and various other surface chemistries enables remarkable materials processing and possible biomedical applications that go well beyond the evolved biological function, as discussed below.

At this point, relatively little is known about the specific composition of the hygroscopic matrix except that it likely comprises various hemicelluloses and pectins (17). The ability of pectin molecules to create hydrogel networks through formation of ionic interactions may be relevant for the hygroresponsive behavior of the viscin tissue; however, this remains to be determined. Indeed, elucidating chemical level structure–function relationships in the viscin tissue will be a major focus of future studies. This will provide a better understanding of the natural material behavior and provide a stronger foundation for realizing bioinspired applications. It will be important to establish the exact chemical composition and structure of the matrix biomolecules, how they are associated with the CMFs, and how they contribute to the distinctive properties of the viscin tissue including its hygroscopic behavior, fiber/film drawing, and adhesion.

### Fiber fusion and hygroscopic contact welding

**Fig. 8.**
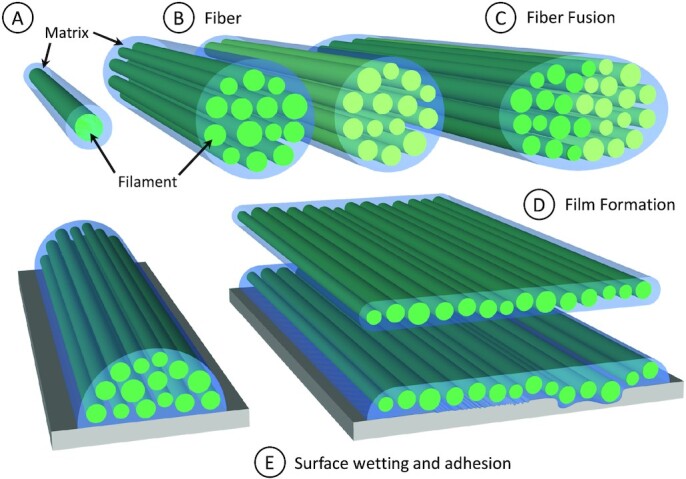
Schematic model of viscin fiber and film formation. (A) Basic building blocks of viscin fibers and films are cellulosic filaments surrounded by a noncellulosic adhesive matrix. (B) Viscin fibers are comprised of filaments glued together by the matrix. (C) Individual viscin fibers can fuse into a single fiber by rehydration of the matrix. (D) Viscin films are comprised of the same building blocks as viscin fibers, but in a different configuration induced through biaxial stretching. (E) Hydrated fibers and films are able to adapt to various surface geometries with different surface shapes, roughness, or waviness, where the matrix allows a structural reorganization of the embedded filaments in the hydrated state and adheres viscin fibers and films firmly in the dried state.

Contact welding behavior under ambient conditions is not a typical property of most thermoplastics, and fusion of 2 separate surfaces, would require bringing a typical thermoplastic near its melting point, and essentially remolding 2 surfaces into 1 (26). Many supramolecular polymer materials, including vitrimers, ionomers, and supramolecular hydrogels, do exhibit welding and self-healing behaviors under ambient conditions based on reversible noncovalent bonding interactions (e.g. hydrogen bonding, metal coordination, and ionic interactions) (27–29); however, these materials are typically extremely soft. In contrast, mistletoe viscin fibers are extremely stiff, yet flexible, and still capable of fusion and self-healing triggered simply by cycling between low and high humidity conditions, resulting in the fusion of multiple fibers (Fig. 2). The mechanical prowess of the fibers can be ascribed to the stiff cellulose; however, we posit that the welding behavior arises from interactions between the swellable hygro-responsive matrix material, which is an integrated component of the viscin, and perhaps even covalently linked to the surface of the cellulose (Fig. 8A–C) (11). Currently, however, very little is understood about the chemical mechanism of welding due to the limited compositional studies performed on viscin from mistletoe species, which only indicate the presence of various hemicelluloses and pectin molecules in addition to cellulose, none of which is unusual for a material that is formed from the primary cell wall (17, 30–32). Nonetheless, the combination of a hygro-responsive adhesive reinforced by stiff cellulosic fibrils provides an exciting bioinspired paradigm for design of high-performance supramolecular composites, combining exceptional mechanical properties with intrinsic self-healing response and capacity for contact welding.

### Film formation

We demonstrated here that mistletoe viscin can be stretched not only into uniaxial fibers (11), but also into free-standing films via application of a multiaxial load and drying. Considering the viscin tissue as a composite of CMFs embedded in a supramolecular hygro- and mechano-responsive matrix, one can envision the film formation process as a force-induced reorientation and uncoiling of viscin cellulose into aligned CMFs along various directions corresponding to local stress/strain fields under multiaxial load. At the same time, the matrix maintains the integrity of the film, holding CMFs together via reversible sacrificial bonds (Fig. 8E). This is supported by the fact that CMFs are oriented along the fiber axis near anchoring points but are less oriented in the film center and may remain as coiled viscin cells. This provides a reservoir of hidden length to further expand the surface area of a dried film in any direction following rehydration, and likely plays a role in preventing film failure under drying stresses.

### Multimaterial adhesion

The natural substrate of the viscin adhesive is the tree bark covering the branches of host species (14, 15); however, we demonstrated that viscin is essentially indiscriminate in terms of what surface chemistry it adheres to, showing adhesion to both biogenic materials such as wood, skin, and cartilage, as well as completely synthetic materials including various plastics, metal alloys, and glass. The adhesive strength measured from lap shear tests of viscin on wood under dry conditions (>2 MPa) are at least 3 times lower than those reported for standard synthetic wood glues based on polyvinyl acetate (PVA) and polyurethane, although reported values vary between studies (33, 34). However, unlike PVA and polyurethane glues, which are primarily petroleum-sourced and slow to degrade (35), mistletoe viscin is comprised of biorenewable and biodegradable molecular components. In addition to wood adhesion, adhesion to skin may also be relevant evolutionarily since adhesion to bird feathers and beaks, which share a keratinous origin similar to mammalian skin (36), also constitutes a seed-spreading mechanism (e.g. a bird will dislodge a seed that has become adhered to its beak onto a branch). Yet, the adherence to various synthetic surfaces that represent both polar and nonpolar surface chemistries is harder to explain from an adaptive standpoint and may simply represent a highly versatile adhesion chemistry.

Currently, the chemical and physical mechanisms of viscin adhesion is completely unknown. Nonetheless, it seems likely that the ability of the viscin to adapt its shape to a surface it is in contact with in the wet state enables mechanical adhesion as the material dries and locks into nano- and micro-scale pores on the surface (e.g. similar to cyanoacrylate glues; Fig. 8D and E). However, specific chemical adhesion mechanisms are also suggested since viscin also binds to freshly cleaved mica, on which surface roughness is negligible and to cartilage, which typically presents substantial challenges for adhesion (37). The ability to stick to both hydrophilic (e.g. mica, biological) and hydrophobic (PTFE) surfaces suggests that viscin adhesion is chemically versatile. This is not unprecedented in the biological world—catechol-based mussel adhesives bind to hydrophilic surfaces via hydrogen bonds, ionic bonds, or metal coordination, and bind to nonpolar surfaces through hydrophobic interactions (6). Notably, catechols have been detected in viscin of certain mistletoe species (32). However, at this point there are no clear connections between adhesion and any aspect of the viscin chemical composition. In addition to chemical adhesion mechanisms, mechanical reinforcement of adhesives can be a critical feature in their performance. As demonstrated with gecko and gecko-inspired adhesion, the compliance of an adhesive is inversely related to the pull-off force (38). In this light, the inclusion of stiff CMFs in the viscin adhesive likely contributes positively to the adhesive prowess of mistletoe viscin as well.

### Biomedical potential for viscin coatings

The demonstration that viscin can adhere to biological tissues including cartilage and skin raises the potential for biomedical applications, especially given the clear need for biocompatible and biodegradable tissue adhesives (39, 40), and the recent rise in literature using cellulose based scaffolds for tissue engineering (41). In particular, the observation that mistletoe viscin can adhere to cartilage is especially remarkable considering the enormous challenges in developing effective adhesives for wet surfaces in biological tissues (37, 42, 43); however, further work is required here. With regards to skin coatings and wound sealants, the processability of viscin into a coating is relatively straightforward, as demonstrated with film formation. No further additives are required—as long as the viscin stays hydrated, the coatings can be formed and applied under ambient conditions. Indeed, the humidity from skin moisture appear to be enough to keep the viscin film pliable and sticky for at least several days even during brief washing; yet, it is easily removable with a bit of friction. However, in terms of potential biomedical applications and reproducibility, it would be important in the future to understand the relationship between processing conditions (e.g. draw speed and film dimensions) and the specific mechanical properties of the films. Additionally, inspired by ancient recipes, simple treatment of viscin from numerous berries with olive or walnut oil, produces a larger, less sticky, smooth film that is comfortable to wear and more flexible, providing fewer restrictions in using viscin-coated hands.

As opposed to currently available nitrocellulose-based wound sealants, the viscin films are sustainable natural products with no synthetic additives, are environmentally friendly, are biodegradable, and even function under wet conditions (suggesting they could function in the presence of bodily fluids such as blood). A crucial point to establish in future studies with regards to potential biomedical applications is the biocompatibility of the viscin tissue, and whether it may contain substances toxic to cells. While no skin irritations were observed in the current study, there are numerous earlier studies that have touted potential anticancer and therapeutic compounds that have been extracted from mistletoe viscin (44, 45), although there is some dispute on this topic in the literature (46, 47). While this potentially adds a further dimension to the possible biomedical applications of these biocoatings, toxicity analysis performed in these studies suggests that some of the compounds affect cells in culture (48).

## Experimental Section

### Material

Whole *V. album* L. ssp. *album* plants with mature berries were collected from apple trees (*Malus domestica*) near Golm, Germany in the winters of 2017/18 and 2018/19. All berries were cut from the mistletoe branches in groups with intact peduncles to maintain the structural integrity of the individual berries. Berries were either used immediately for experiments or were flash frozen by collecting berries in 50 ml falcon tubes sealed with parafilm and submerged into liquid nitrogen for 5 min. Falcon tubes were stored at −20°C and single berries were thawed for later use. The freeze–thaw process did not result in noticeable differences of the fiber or film forming ability. For the mechanical characterization of the adhesive properties, fresh berries were harvested in the winter of 2021 and used the same day.

### Methods

**Fiber Drawing**. Viscin fibers were drawn mechanically by hand using tweezers following the previous work of Horbelt et al. (11) (see Figure S1 [Supplementary Material] for a more detailed description of the fiber drawing process). Freshly drawn adhesive fibers were either used in the still hydrated state or attached to a laboratory stand and allowed to dry at ambient conditions under the weight of the attached seed to be used later.

**Film drawing**. Viscin was isolated mechanically from the mistletoe berry and immediately drawn into films with the help of tweezers as long as the material was hydrated and then allowed to dry under ambient conditions for further analysis. A detailed description of different methods to process the viscin into films can be found in the SI (Figures S2 and S3, Supplementary Material).

**Light microscopy and polarized light microscopy (PLM)**. Samples were investigated with a digital microscope (Keyence VHX-S550E) equipped with a universal objective (VH-Z100UR) under crossed polarizers with optional use of a red full-wave retardation plate for PLM. Videos were recorded with a framerate of 15 Hz and a camera resolution of 1,600 × 1,200 pixels.

**ESEM**. Samples were investigated in an ESEM in a low-vacuum mode (FEI Quanta FEG 600). Images were obtained at an acceleration voltage of 4–5 kV using a secondary electron detector.

**WAXS**. Synchrotron based WAXS experiments were conducted at the mySpot beamline (Paris et al. (49)) at the BESSY II synchrotron radiation facility (Helmholtz-Zentrum Berlin, Adlershof). Viscin films were prepared as described above and mounted perpendicular to the incident X-ray beam. The diameter of the incident beam was ∼50 µm. Line scans across the films were performed with a step size of 50 µm and a measurement time of 60 s per scan point. The wavelength of the incident beam was 0.082656 nm. WAXS patterns were collected with a 2D CCD detector (Rayonix MAR Mosaic225) with a total area of 3,072 × 3,072 pixels and a pixel size of 73.2 µm × 73.2 µm at a sample-to-detector distance of ∼30 cm. 2D scattering patterns were further processed and analyzed with dpdak v.1.3, an open source XRD analysis tool (49).

**Mechanical characterization of the adhesive strength**. Lap shear specimen were prepared to measure the tensile shear strength. Lap joints were prepared using beech plywood strips (L × W × T dimensions: 100 mm × 25 mm × 2mm) with an overlap joint length of 12.5 mm. For each specimen, the viscin from single berries was isolated mechanically with tweezers as described in the SI section and equally spread over the designated overlapping joint area. The 2 plywood strips were carefully positioned and clamped with a custom-built device, where each specimen was compressed for 30 min. Afterward, specimens were stored over night at lab conditions (21°C and 30% RH) in a clamping device applying moderate compression. After drying, overnight samples were released from the clamping device. In total, 1 group was tested after 24 h while 2 other groups were conditioned for 6 further days prior to testing at either 21°C/30% RH and 21°C/60% RH. Shear lap tests were performed on a Zwick universal testing machine (zwickiLine Z2.5) equipped with a load cell with a maximum capacity of 2.5 kN. Test speed was set to 10 µm s^–1^ and 15 successful tests were carried out for each group.

## Authors' Contributions

All authors planned the experiments. N.H. performed the experiments. All authors interpreted the results and wrote the manuscript.

## Supplementary Material

pgac026_Supplemental_Files

## Data Availability

Data sharing plan: all data are available in the main text or the supplementary materials.
