# Supplementary material for: Mistletoe viscin: a hygro- and mechano-responsive cellulose-based adhesive for diverse material applications
Source: PNAS Nexus. 2022 Mar 16;1(1):pgac026. doi: 10.1093/pnasnexus/pgac026 (PMC9802232; doi:10.1093/pnasnexus/pgac026)
Supplement: pgac026_Supplemental_Files [file pgac026_supplemental_files.zip › PNASNEXUS-PNASNEXUS-2021-00123-s01.docx]

**Supporting Information**

**Mistletoe Viscin: A Hygro- and Mechano-Responsive Cellulose-Based Adhesive for Diverse Materials Applications**

Nils Horbelt^1^, Peter Fratzl*^1^, Matthew J. Harrington*^1,2^

^1^Dept. of Biomaterials, Max Planck Institute of Colloids and Interfaces, Potsdam 14424, Germany

^2^Dept. of Chemistry, McGill University, 801 Sherbrooke Street West, Montreal, Quebec H3A 0B8, Canada

Email: [peter.fratzl@mpikg.mpg.de](mailto:peter.fratzl@mpikg.mpg.de), [matt.harrington@mcgill.ca](mailto:matt.harrington@mcgill.ca)

**Manual Viscin Fiber Drawing.** Mistletoe berries were harvested with the berries still attached to the peduncle to maintain the structural integrity of the berries. As described in a time series of images in Figure S1, holding the peduncle in one hand and pulling the berry with the other hand, the berry can be removed from the peduncle. This leads to a small opening at the former connection point. By mimicking the behavior of some mistletoe feeding birds, it is possible to squeeze the seed out of the berry by applying a gentle pressure onto the berry with one’s fingertips. The seed slips through the opening which is widened as the seed passes the opening (Fig. S1B). Thereby the seed turns by 180° (Fig. S1C). By grabbing the sticky seed with tweezers it can be further pulled out of the remaining berry which results in the formation of two individual viscin fibers each emerging from the two mirrored VCBs, connecting the seed with the remains of the berry (Fig. S1D). Initially these fibers were only a few mm to cm long, extremely sticky and highly extensible. Upon further drawing, they readily extend into thin fibers (diameter 20 - 100 µm) with a final length of more than 1 m each. If the two individual fibers come into contact during the drawing process, they adhere and macroscopically fuse into a single fiber.

**Figure S1.** **Time series images of the manual drawing of mistletoe viscin fibers.** A) Removing the berry from the peduncle. B) Compressing the berry will press the seed out of the surrounding berry pericarp near the former peduncle. C) The exposed sticky seed can be grabbed with tweezers. D) Pulling the seed away from the berry leads to the initial formation of two viscin fibers. E) Pulling the seed further away leads to an elongation of the fibers. On one side the fibers emerge from two viscin cell bundles connected to the seed. F) On the other side they emerge from viscin cell clusters which are connected to the surrounding fruit flesh. The fruit skin can often be readily removed from the flesh.

**Fusing of Viscin Fibers.** A viscin fiber was drawn manually as described above. The dried fiber was cut into short segments of ~2 cm. The fiber segments were glued onto a foliar frame with super glue, leading to a free fiber length of ~5 mm. The glued fiber and the foliar frame were cut in the center with a razor blade. The two loose fiber ends were moved together again so that they aligned parallel with an overlap of ~1 mm. Then the fiber ends were exposed to a stream of saturated water vapor for 90 seconds generated by a humidifier which leads to a permanent fusion of the former loose fiber ends. The fusing process was observed with PLM video imaging using a digital microscope as described below. The fused fiber segment was cut with a razor blade perpendicular to the long axis and imaged with ESEM as described below.

**Contact Welding of Viscin Fibers.** Viscin fibers were drawn manually and dried as described above before they were cut into short segments of ~2 cm. The fibers were glued onto a cardboard frame spanning a circular window with a diameter of ~16 mm as follows: A first layer of parallel fibers was glued onto the cardboard in a horizontal string pattern. Each fiber was placed in position with the loose ends on the cardboard. The tip of the index-finger was moistened and gently pressed onto one loose fiber end for a ~2 s. Afterwards the fingertip is moistened again and pressed on the other fiber end. This was repeated for all horizontal fibers. Analogously a second layer of fibers was deposited in a vertical string pattern creating a fiber mesh. In the same way multiple further layers can be added (Figure S2). The fiber mesh was investigated with PLM and ESEM. Then the mesh was exposed to a stream of saturated water vapor for 90 s generated by a humidifier which leads to a self-welding of all crossed fibers at the fiber nodes. After drying under ambient conditions for 5 minutes the fused fibers were investigated again with PLM and ESEM.

Alternatively, fiber contact welding can be demonstrated on freshly drawn fibers which were not allowed to dry but immediately glued onto a cardboard frame as follows: One end of the freshly drawn fiber was attached to an arbitrary point of the cardboard, exploiting the natural adhesive character of the fiber which instantly adheres firmly to the cardboard. The loose end of the fiber is drawn over the cardboard window towards an arbitrary edge where the fiber is laid down which again instantly adheres the drawn fiber along the small segment which is contact with the cardboard. The loose end of the fiber is again drawn over the cardboard window towards an arbitrary edge of the cardboard where the fiber is laid down. Repeating this procedure quickly allows one to generate a fiber mesh of any desired pattern. When the fiber comes in contact with previously laid down segments the crossed fiber segments instantly adhere and become fused at the fiber nodes. The mesh quickly dries under ambient conditions after which it was cut free along the edges of the cardboard frame with scissors or a razor blade. The mesh could be handled with tweezers. Six meshes were arranged as a cube where a large number of free fiber ends along the edges of the individual meshes was used to create a temporary interlocking. The cube was exposed to a stream of saturated water vapor for 60 s generated by a humidifier leading to the welding of the numerous crossed fiber segments along the edges of the cube.

**Figure S2. ESEM studies of the self-welding behavior of multilayered viscin fibers.** A) 3 staggered layers of dried viscin fibers successively arranged perpendicular to the adjacent layer. Images A-D are oblique views taken at an angle of 25°. B) Detail of (A) showing the circular shaped fibers are not connected. C) Self-welded viscin fibers after rehydration due to short time exposure to saturated water vapor. Scale bar: 500 µm. D) detail of (C) showing all 3 fibers welded together. The formerly independent parallel bottom and top layered fibers fused along their entire length. The center fiber gets fused to the adjacent fibers at the fiber crossings in a sandwich-like structure. The fibers become deformed after rehydration and appear flatter and broader than the original circular fibers which is particularly pronounced for the center fiber towards the nodes.

**Making Films from Mistletoe Viscin.** Here, we describe the simplest way to make a viscin film: first the peduncle of the berry has to be pulled off. By gently compressing the berry between two fingertips the seed is pushed out of the bottom opening of the berry skin. Then the remaining berry is grabbed with two fingers of both hands each and a firm pressure is applied on the berry until it bursts (Fig. S3A). The mucilaginous remaining viscin within the berry is released which will instantly adhere to the fingertips. Now both hands and both fingertips can be moved apart by a few centimeters inducing the formation of a semi-transparent 2-dimensional film which is firmly connected to the fingertips and the seed (Fig. S3B+C). The freshly formed film area can be instantly decreased again by simply moving the fingertips and/or hands closer to each other and can be increased again the other way around. Not only the size but also the film shape can be dynamically changed by independently moving the fingers or even rotating the hands. A freshly formed adhesive viscin film can be applied onto a wide range of material surface and readily adapts to various surface shapes and surface topographies, e.g. curved or flat, rough or smooth surfaces.

**Figure S3. Simple viscin film formation.** A) After the peduncle is removed and the seed is released from the berry, the remaining berry is compressed between the fingertips. B) With the viscin firmly adhering to the skin the viscin can be stretched into a transparent film by moving both hands apart. C) The film readily extends upon further stretching.

**Drawing dimensionally stable free standing 2D films.** Single berries were placed into a small glass Petri dish with a diameter of 5 cm. A large incision was made on the top of the berry with a razor blade. The seed was carefully taken out of the berry with fine tweezers through this opening in the fruit skin and was put aside in the Petri dish close to the remaining berry (Fig. S4A). Then the viscin was carefully isolated by hand using tweezers with a broad tip in a stepwise procedure as follows: The tweezers were used to grab the viscin cell clusters, located on the inside of the fruit flesh and pull them out of the berry with a short and slow movement to reduce the mechanically induced formation of fibers as best as possible. The viscin was deposited on the bottom of the Petri dish next to the seed. The last two steps were repeated until the viscin inside the berry was depleted (Fig. S4B). The remains of the fruit skin were grabbed with tweezers and glued to the edge of the Petri dish exploiting the natural adhesive properties of the viscin. Then the seed was glued to the opposite side of the Petri dish and the isolated viscin was carefully lifted off of the bottom, which leads to the formation of a thick viscin strand between the seed and the fruit skin (Fig. S4C). To form a triangular shaped film, one can grab the strand at any location and pull it towards an arbitrary edge of the Petri dish where it can be fixed by pressing the newly formed pointed end of the film onto the glass for ~30 s (Fig. S4D-E). While pressing, the film must be gently kept under tension to maintain the film shape because otherwise the hydrated viscin film promptly collapses into three thick strands. To form a quadrangular shape, one can grab another film edge and pull it towards any edge of the Petri dish and fix it (Fig. S4E-F). A quick repetition of this procedure allows one to form various 2-dimensional polygons as long as the viscin remains hydrated. In order to achieve more complex 3-dimensional film geometries, one can select different height levels for the exterior connection points. The resulting film spans the area between the connecting points like a stretched tarpaulin. The viscin films quickly dry under ambient conditions. The thinner center area of the films dries faster than the thicker edges. After drying the dimensionally stable films can be manipulated with tweezers and cut into any shape with tools such as razor blades, knifes or scissors. Tapered ends can also be simply sheared off with a blunt tool.

**Figure S4. The making of free standing viscin films in a Petri dish.** A) After making an incision on top of the berry skin, the seed is removed from the berry and put closely aside. B) Using broad tipped tweezers the viscin is carefully pulled out of the berry with slow and short strokes. The viscin is deposited in the center of the Petri dish. C) Exploiting the natural adhesive properties the seed is attached to one edge of the Petri dish, the remaining fruit skin to an opposite edge. Both seed and fruit skin are connected via a thick strand of the isolated viscin. D) The viscin strand is gently grabbed with tweezers and pulled towards the left edge of the Petri dish. E) The viscin was attached to a third connection point which leads to the formation a triangular shaped viscin film. The edge of the freshly formed film is grabbed again and pulled towards an edge on the right. E) A quadrangular viscin film is being formed.

**Viscin multimaterial adhesion tests.** Viscin was isolated from single berries as described above for the making of viscin films where the seed and the fruit skin were cut off at both ends of the isolated viscin. The viscin of a single berry was deposited on the top surface of a cylinder comprised of a specific material and drawn into a fiber which was attached to a horizontal steel bar (exploiting the natural viscin adhesiveness) of a laboratory stand placed above the cylinder. In this manner, 10 viscin fibers were made connecting the metal bar with 10 aligned cylinders each consisting of a different material: brass, aluminium, stainless steel, quartz glass, polytetrafluoroethylene (PTFE), high density polyethylene (HDPE), polycarbonate (PC), polyamide (PA), polypropylene (PP) and Beech wood. Each cylinder had a diameter of 10 mm and a weight of ~10 g. The viscin was allowed to dry for 2 h until the metal bar was lifted by 10 cm so the dried viscin fibers were carrying the load of the free hanging cylinders.

To test the adhesive properties of viscin in combination with cartilage a pork knee joint was used from a fresh pork leg, bought from Fleischerei Domke, Berlin. The cartilage from the knee joint was isolated from the surrounding tissue. Viscin and seed were extracted as described for the making of viscin films and attached to the freshly exposed cartilage tissue.

**Preparing wound sealings and skin coatings from viscin.** The viscin of multiple individual berries can be mixed. Therefore, the viscin can be isolated and collected in a Petri dish as described above after which the isolated material can be cut free from the seed on one end and from the fruit flesh and skin on the other end using a razor blade or scissors. The viscin of a second berry can simply be added to the previously isolated viscin during which the two viscin units instantly adhere and fuse into a sticky mass. By adding viscin of more berries the total mass of viscin can be increased at will. The mixing can be enhanced by slowly stirring the viscin mass with a spatula. The sticky coherent mass can be picked up from the Petri dish either by hand or with tools like tweezers or spatulas. The bulk viscin can then be deposited onto the skin region it is supposed to seal. Starting from the initially covered region the viscin can be readily distributed by shearing the material along its edges where it instantly adheres to the newly covered area. Within several minutes the viscin dries into a transparent film. If the viscin mass is not sufficient enough to cover the desired surface, the remaining area can simply be covered by adding isolated viscin from more berries. This can be done with freshly applied viscin, but also when the viscin sealing already partly or even completely dried. Alternatively, the freshly mixed viscin can be manually drawn into films as described above for single berries which can then directly be applied onto the skin. Dried films can readily be peeled or rubbed off of the skin again, leaving no visible traces.

Following remarks about ancient recipes for the use of viscin for making birdlime and skin coatings, viscin was isolated and mixed as described in the main text and then submerged in commercially available vegetable oil, such as olive oil (de Cecco, Italy), rapeseed, linseed or walnut oil (Kunella Feinkost, Germany) for 5 minutes including gentle stirring with a spatula. Afterwards the coherent viscin mass was removed from the oil bath and the oil covered viscin is kneaded by hand for one minute. The resulting viscin-oil mixture was applied to human skin, in the same manner as the oil free viscin described above. The applied viscin dried into a transparent film within 10 minutes.

Within this work the author used his own hands to test the viscin adhesive properties on live skin tissue. However, tests for the potential use as a wound sealant were performed on porcine skin from a pork leg. A fresh pork leg was purchased from Fleischerei Domke in Berlin and used the same day. Six parallel incisions were made with a razor blade. The incisions were ~20 mm long and ~5 mm deep with a gap of ~10 mm to the neighboring incision. In an alternating sequence three incisions were treated with viscin and the remaining three incisions were taken as a reference. Fresh viscin was isolated from one single berry for each incision and distributed as a thin layer onto the porcine skin. The viscin was allowed to dry for 1 h until the incisions were mechanically loaded by stretching the skin perpendicular to the long axis of the incisions.

**Supporting Video S1.** *In situ* PLM video demonstrating the fusion of viscin fibers under humidity cycling.
